# Assessment of visual fixation in vegetative and minimally conscious states

**DOI:** 10.1186/1471-2377-14-147

**Published:** 2014-07-16

**Authors:** Haibo Di, Yunzhi Nie, Xiaohua Hu, Yong Tong, Lizette Heine, Sarah Wannez, Wangshan Huang, Dan Yu, Minhui He, Aurore Thibaut, Caroline Schnakers, Steven Laureys

**Affiliations:** 1 International Vegetative State and Consciousness Science Institute, Hangzhou Normal University, 310036 Hangzhou, China; 2 Cyclotron Research Centre and Neurology Department, Coma Science Group, University and University Hospital of Liege, Liege, Belgium; 3 Wujing Hospital of Hangzhou City, Hangzhou, China; 4 Huzhou Center Hospital, Huzhou, China

**Keywords:** Disorders of consciousness, Vegetative state, Unresponsive wakefulness syndrome, Minimally conscious state, Visual fixation

## Abstract

**Background:**

Visual fixation plays a key role in the differentiation between vegetative state/unresponsive wakefulness (VS/UWS) syndrome and minimally conscious state (MCS). However, the use of different stimuli changes the frequency of visual fixation occured in patients, thereby possibly affecting the accuracy of the diagnosis. In order to establish a standardized assessment of visual fixation in patients in disorders of consciousness (DOC), we compared the frequency of visual fixation elicited by mirror,a ball and a light.

**Method:**

Visual fixation was assessed in eighty-one post-comatose patients diagnosed with a MCS or VS/UWS. Occurrence of fixation to different stimuli was analysis used Chi-square testing.

**Result:**

40 (49%) out of the 81 patients showed fixation to visual stimuli. Among those, significantly more patients (39, 48%) had visual fixation elicited by mirror compared to a ball (23, 28%) and mirror compared to a light (20, 25%).

**Conclusion:**

The use of a mirror during the assessment of visual fixation showed higher positive response rate, compared to other stimuli in eliciting a visual fixating response. Therefore, fixation elicited by a mirror can be a very sensitive and accurate test to differentiate the two disorders of consciousness.

## Background

At present, there are many behavioral scales available for the evaluation of visual fixation in patients in post-comatose states. These disorders of consciousness (DOC) include VS (now also coined unresponsive wakefulness syndrome) [1], and MCS [2]. Indeed, the scales all use different stimuli to assess visual fixation: the Coma Recovery Scale-Revised (CRS-R) recommends using a brightly colored or illuminated object. The Sensory Modalities Assessment and Rehabilitation Technique (SMART) uses a photo of a baby. The Disorders of Consciousness Scale (DOCS) uses an object (e.g., ball). The Coma/Near Coma Scale employs flashes of light. The Wessex Head Injury Matrix (WHIM) uses a person, and the Western Neuro Sensory Stimulation Profile (WNSSP) only observes spontaneous eye contract (for references see review by Majerus and colleagues [3]).

The CRS-R is at present the most sensitive tool to differentiate between the different states of (un) consciousness [4]. However, visual behavior has been shown to still differ greatly with different stimuli [5]. Vanhaudenhuyse et al. used a mirror and other neutral stimuli to assess visual pursuit in patients with DOC, and showed that a mirror provokes visual pursuit more frequently in patients, due to the self-referential value [6]. Specifically, some patients showed visual pursuit when using a mirror, but not with other stimuli such as a colored ball or a bright light. To our knowledge, the increased effect of a mirror has not yet been investigated in visual fixation. Hence, in this study we choose the visual stimuli described in the CRS-R Administration and Scoring Guidelines: a yellow ball (as brightly colored object), a light (as illuminated object) and a mirror (as self-referential object) to induce visual fixation in patients with DOC. We will compare the ability of different stimuli in eliciting visual fixation in order to optimize the stimulation during the assessment of consciousness.

## Method

Eighty-one patients recovering from coma were recruited from Wujing Hospital of Hangzhou City, Hangzhou, China, and were free of sedative drugs. Each patient was assessed in a sitting position. If patients exhibited sustained eyelid closure and/or stopped following commands for a period of at least one minute, a standardized arousal facilitation protocol (i.e., deep pressure stimulations from the facial muscles to the toes) was employed in order to prolong the time the patients maintained aroused, and this protocol was re-administered if patients showed sustained eye closure again or behavioral responsiveness ceased despite sustained eye opening [7]. Visual fixation was evaluated through the standardized methodology as described in the CRS-R [7]. In brief, a visual stimulus (i.e., a mirror (round, diameter = 15 cm), a ball (yellow, diameter = 6 cm) and a continuous burning light (power = 1.2w)) was presented by the experimenter in front of the patient’s face (15–20 cm) and then rapidly moved above and below the horizontal midline, as well as to the right and left of the vertical midline. Thus the stimulus moved once in each direction (4 trials). The order of presentation was randomized using a “random number” procedure in Excel. Visual fixation is defined as a movement of the eyes from the initial fixation point with a re-fixation on the new target location for more than 2 seconds. At least 2 episodes of fixation are required for the scoring of visual fixation. Differences between fixation as assessed by mirror, ball and light were measured using Chi-square test. Results were considered significant at p < 0.01. Eye movements were observed before administration of stimuli to avoid scoring of spontaneous movements. For example, for subjects with roving eye movements the stimuli were presented in a manner unrelated to pre-existing spontaneous eye movements. When any doubt existed, the movement was not scored. Patients’ visual reaction was visually judged by one experienced assessor who was blinded to diagnosis (e.g., did not do CRS-R assessment), and was unaware of the hypothesis of this study, and the same examiner conducted the trials in all patients. Clinical diagnosis was made according to the Aspen work group criteria for disorders of consciousness [2] and based on the CRS-R assessments [7] performed by two trained and experienced neuropsychologists. The study was approved by the Ethics Committee of Hangzhou Normal University and Wujing Hospital which complies with the Code of Ethics of the World Medical Association (Declaration of Helsinki). Informed consents were obtained by the patient’s legal surrogates.

## Result

**Figure 1:**
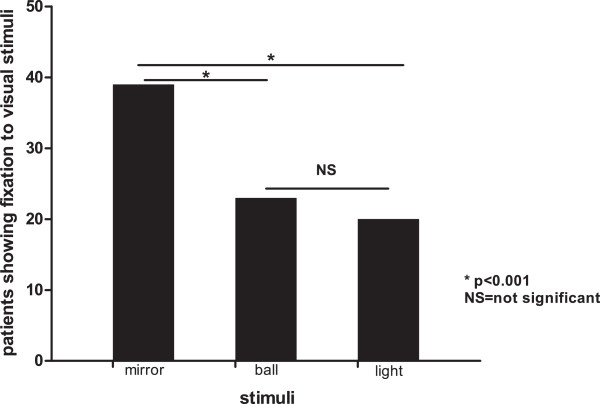
**Visual fixation.** Number of patients in VS/UWS and MCS showing fixation to visual stimuli (n = 40) as a function of the employed stimulus (mirror, ball and light).

Of the 81 patients (69 males; mean age 45 (SD17) years), 38 (47%) were diagnosed as in a VS/UWS and 43 (53%) in a MCS. Mean time between injury and assessment was 9 months (SD15). Etiology was traumatic in 59 (73%) and non-traumatic in 22 (27%) patients (Table 1). 40 (49%) out of the 81 patients showed visual fixation, which were all diagnosed as in a MCS. Of the patients who showed visual fixation, 39 (48%, 28 traumatic) showed fixation to a mirror, 23 (28%, 15 traumatic) showed fixation to a ball, and 20 (25%, 15 traumatic) showed fixation to light. Except one, no patient showed fixation to the ball or the light without the mirror. The global value of the observed Chi-2 statistic between the mirror, ball and light was 27.22, and p < 0.001. When doing the local comparisons between the three stimuli, the mirror elicited significantly more visual fixation compared to the two other stimuli (p < 0.001), while the difference between the frequency of visual fixation assessed by a ball and a light was not significant (Figure 1). The occurrence of visual fixation had no significant relationship with etiology or time since insult (p > 0.05). The overall behavioral responsiveness as assessed by the CRS-R total score tended to be higher when patients fixated on all three stimuli (n = 17) compared to no fixation (n = 41) (Table 1). In fact, patients showing a response to the mirror, the ball and the light had a mean CRS-R total score 9.6 whereas patients showing no fixation to any of the stimuli had a mean score 4.8, and patients having fixation to two (n = 8) or one (n = 15) stimuli showed intermediate mean CRS-R total scores of respectively 9.3 and 8.3. Correlation analysis showed that the rank correlation coefficient between the number of stimuli fixated by patients and the CRS-R total score was 0.743, p < 0.001. Multiple regression analysis did not show an effect of sex or age and the p values of the partial regression coefficient were 0.174 for sex and 0.553 for age.

**Table 1:** Clinical data of patients in VS/UWS and MCS

**Patient**	**Gender**	**Aetiology**	Time since injury**	**CRS-R total score**	**Visual subscale score**	**Stimulation elicited positive visual fixation**
MCS1	male	trauma	2	6	0	none
MCS2	male	trauma	6	6	1	none
MCS3	male	Non-trauma	12	8	1	none
MCS4*	male	trauma	3	5	3	mirror
MCS5*	male	trauma	4	5	3	mirror
MCS6*	female	trauma	1	6	3	mirror
MCS7*	male	Non-trauma	2	7	3	mirror
MCS8*	female	trauma	5	7	3	mirror
MCS9*	male	trauma	5	7	3	mirror
MCS10*	male	trauma	4	7	3	mirror
MCS11*	female	trauma	9	8	3	mirror
MCS12*	male	Non-trauma	119	8	3	mirror
MCS13*	male	trauma	33	9	3	mirror
MCS14*	male	trauma	3	9	3	mirror
MCS15*	male	trauma	7	10	4	mirror
MCS16*	male	trauma	4	13	3	mirror
MCS17*	male	trauma	7	16	4	mirror
MCS18	male	trauma	5	7	2	ball
MCS19	male	trauma	8	6	3	Mirror and ball
MCS20	male	trauma	9	7	3	Mirror and ball
MCS21	male	trauma	5	7	3	Mirror and ball
MCS22	male	trauma	27	8	3	Mirror and ball
MCS23	female	trauma	4	10	2	Mirror and ball
MCS24	male	Non-trauma	1	10	3	Mirror and light
MCS25	male	trauma	8	11	3	Mirror and light
MCS26	male	trauma	10	15	5	Mirror and light
MCS27	male	trauma	2	6	3	All three
MCS28	male	Non-trauma	2	6	4	All three
MCS29	male	trauma	3	7	3	All three
MCS30	female	trauma	37	8	3	All three
MCS31	male	Non-trauma	11	8	3	All three
MCS32	male	Non-trauma	2	8	2	All three
MCS33	male	trauma	12	8	3	All three
MCS34	female	Non-trauma	4	9	3	All three
MCS35	male	trauma	47	9	3	All three
MCS36	male	Non-trauma	31	9	3	All three
MCS37	female	trauma	12	10	3	All three
MCS38	female	Non-trauma	3	10	3	All three
MCS39	male	trauma	3	10	4	All three
MCS40	male	trauma	4	12	3	All three
MCS41	male	Non-trauma	12	13	4	All three
MCS42	male	trauma	6	15	4	All three
MCS43	male	Non-trauma	30	16	4	All three
VS1	male	trauma	5	1	0	none
VS2	female	Non-trauma	5	2	0	none
VS3	male	Non-trauma	11	2	0	none
VS4	male	trauma	3	2	0	none
VS5	male	trauma	9	2	0	none
VS6	male	trauma	1	3	0	none
VS7	male	Non-trauma	1	3	0	none
VS8	male	Non-trauma	5	3	0	none
VS9	female	trauma	1	3	0	none
VS10	male	trauma	6	3	0	none
VS11	male	trauma	7	3	1	none
VS12	male	trauma	3	4	1	none
VS13	male	trauma	8	4	0	none
VS14	male	trauma	4	4	1	none
VS15	male	trauma	8	4	0	none
VS16	male	trauma	6	4	1	none
VS17	male	trauma	3	4	1	none
VS18	male	Non-trauma	10	5	0	none
VS19	male	trauma	14	5	0	none
VS20	male	Non-trauma	3	5	0	none
VS21	male	trauma	5	5	0	none
VS22	male	trauma	9	5	0	none
VS23	male	trauma	8	5	1	none
VS24	male	trauma	4	6	0	none
VS25	male	trauma	3	6	0	none
VS26	male	Non-trauma	5	6	1	none
VS27	male	trauma	17	6	0	none
VS28	male	Non-trauma	5	6	1	none
VS29	male	Non-trauma	9	6	0	none
VS30	male	trauma	3	6	1	none
VS31	female	trauma	3	6	0	none
VS32	male	trauma	4	6	0	none
VS33	male	trauma	5	6	0	none
VS34	male	trauma	2	7	1	none
VS35	male	Non-trauma	2	7	0	none
VS36	male	trauma	12	7	1	none
VS37	female	trauma	5	7	1	none
VS38	male	trauma	5	7	1	none

*MCS patients who only showed visual fixation to a mirror but not to a ball and a light.

**Time since injury in months.

Fourteen patients (MCS 4 to 17 labeled with “*” in Table 1) showed visual pursuit but no fixation when assessed with only a bright colored or illuminated object as stated in the CRS-R operation. However, they showed visual fixation when a mirror was presented.

## Discussion

The aim of the present study was to determine whether the assessment of visual fixation in patients recovering from coma is influenced by the choice of visual stimuli. We used a mirror, a colored ball and a light to assess visual fixation and compared the occurrences elicited by different stimuli. Our results showed that indeed the frequency of visual fixation in patients with DOC is related to the visual stimulus used. MCS patients tended to fixate significantly more on their own reflection (48%) as compared to fixation on a brightly colored (28%) and illuminated object (25%). As mentioned by Vanhaudenhuyse et al. [6], auto referential stimuli capture our attention and give rise to a sense of self-awareness in everyday social interactions. This is reflected in the cocktail party phenomenon, which refers to the fact that one’s own name can easily catch his/her attention in a cacophony of conversations and background noise [8]. Similarly, one’s own name induced a larger response in patients with DOC as compared with neutral auditory stimuli [9]. Seeing one’s own face also has similarly strong attention grabbing properties [10]. Functional imaging has previously shown activation of anterior and posterior midline structures (i.e., mesiofrontal and precuneal cortices) during presentation of one’s own face in healthy volunteers [11]. Interestingly, these areas are amidst the most metabolically impaired in patients in a vegetative state [12], possibly explaining why they cannot visual follow or fixate.

The occurrence of visual fixation seems to be related to the patient’s overall behavioral profile. VS/UWS patients showed no visual fixation, the more stimuli the patient showed fixation to the higher the CRS-R score was (i.e., fixation to 0, 1, 2 and 3 stimuli, obtained a score of 4.8, 8.3, 9.3 and 9.6, respectively). Due to clinical limitations we could not use an eye-tracker, to more objectively measure fixation. However, our results overlap with a study that used an eye tracking machine to assess visual tracking behavior and was able to differentiate MCS from VS/UWS [13]. In our group, only three out of 43 patients in MCS failed to show visual fixation. Neurological assessment showed that one of these 3 patients failed to eye blink to threat, indicating impaired brainstem reflexes. The remaining two patients had intact brainstem reflexes and reproducible but inconsistent command following, which could be explained by a visual impairment [14].

Our results showed that except one, all of the patients who responded to the ball and light also responded to the mirror. The one patient that showed visual fixation to the ball but not to the mirror or light. In this case, presentation of the mirror was the last stimulus and hence the fluctuating levels of arousal, generally observed in MCS, might account for the fixation on a ball in the absence of fixation on the mirror. One could argue that the order of presentation could have impacted the level of response of our patients. However, the stimuli were presented in a randomized order and not in a fixed order; suggesting that the high rate of response observed using the mirror cannot be explained by the order of presentation.

According to current guidelines for behavioral assessment, visual fixation differentiates unresponsive from minimally responsive [2]. The assessment of visual fixation according to these guidelines (e.g., use an object), failed to show fixating behavior in many of our subjects, while higher cognitive functions existed. This is inconsistent with the structuring principle of CRS-R. However, this phenomenon disappears when a mirror is used for the assessment of visual fixation. Therefore, using a mirror for visual fixation is sensitive and accurate in the differentiation between MCS and VS/UWS. These results are in line with earlier work emphasizing the use of a mirror in the evaluation of visual following [6]. Thus, we advise to use a mirror for the assessment of visual fixation, especially when visual following is observed using other stimuli. The clinical implications of our findings are important. More than 35% of the MCS patients with visual fixation only fixate on a mirror (and not on other objects). Hence, these patients would be misdiagnosed as being unresponsive when other sensory modalities fail to elicit a behavioral response. Our findings emphasize the importance of using a mirror when evaluating visual fixation in post-comatose states.

Although the use of a mirror is a strong and sensitive stimulus to elicit visual responses due to its self-referential value, we have not tested all possible stimuli as advised in other existing scales. As the CRS-R is currently considered as the gold standard in the assessment of disorders of consciousness [4], we chose the visual stimuli described in the guidelines (i.e., a colored ball or a bright light). We here advise to use a mirror in the assessment of visual fixation; however we cannot make any conclusions about the sensitivity and accuracy of other stimuli, like those mentioned in other existing scales. Future research should therefore focus on including more visual stimuli. Besides, in our study, we did not have the opportunity to collect follow-up data. Nevertheless, this could be done in the future. Indeed, as considering visual fixation as a sign of consciousness has been previously debated [15], future studies should investigate if patients showing visual fixation (detected using a mirror) present a more frequently full recovery of consciousness as compared to patients who do not show such behavior.

One could argue that using an eye-recorder could standardize the way in which the data are collected. However, the use of such method is difficult in our population. Patients recovering from coma often have brainstem lesions or ocular trauma that may affect eye-movements and complicate the use of an automated recording (often validated in healthy volunteers).

## Conclusions

This study emphasizes the use of a mirror during the assessment of visual fixation, as shown by the higher positive response rate of the mirror, compared to other stimuli in eliciting a visual fixating response, adding to previous studies the importance of using auto-referential stimuli in patients with disorders of consciousness (i.e., the use of a mirror in the assessment of visual tracking and one’s own name in the assessment of localization to sound [6,9]).

## Abbreviations

VS/UWS: Vegetative state/unresponsive wakefulness syndrome; MCS: Minimally conscious state; CRS-R: Coma Recovery Scale-Revised; DOC: Disorders of consciousness.

## Competing interests

The authors declare that they have no competing interest.

## Authors’ contributions

YN, XH, YT, WH and DY collected data and managed the patients; HD and YN performed data analyses; SL and HD designed the study; YN, LH, SL and HD wrote the paper. SW, CS, AT and MH revised the manuscript for important intellectual content. HD and YN contributed equally to the study. All authors discussed the results and commented on the manuscript. All authors read and approved the final manuscript.

## Pre-publication history

The pre-publication history for this paper can be accessed here:

http://www.biomedcentral.com/1471-2377/14/147/prepub
